# Culturally inclusive teaching in diverse classroom settings in Chinese kindergartens: a qualitative “context–methods–outcomes” model

**DOI:** 10.3389/fpsyg.2026.1772144

**Published:** 2026-02-12

**Authors:** Yue Li, Mingli Fan

**Affiliations:** 1 Department of Preschool Education, Shijiazhuang Preschool Teachers College, Shijiazhuang, China; 2 Education Department, Hebei University, Baoding, China

**Keywords:** culturally inclusive teaching, education for sustainable development, educational equity, kindergarten teachers, social sustainability, Sustainable Development Goal 4 (SDG 4)

## Abstract

**Purpose:**

Grounded in the framework of Sustainable Development Goal 4 (SDG 4), this study explores culturally inclusive teaching practices among Chinese kindergarten teachers in multicultural classrooms, identifies core instructional methods and their influencing conditions, and constructs a “context–method–outcome” theoretical model capable of explaining the internal logic of these practices. However, existing research remains insufficient in developing empirically grounded and practice-oriented models to explain how culturally inclusive teaching is implemented at the kindergarten level within the Chinese cultural context.

**Method:**

A qualitative research design was adopted, involving semi-structured interviews with 12 teachers and 8 parents from four kindergartens across different regions of China, as well as classroom observations and relevant documentary materials. Systematic analysis was conducted through open coding, axial coding, selective coding, and theoretical integration.

**Findings:**

This study identified six categories of core teaching methods, and integrated three overarching dimensions: communicative and cognitive support, emotional support and identity construction, and social–environmental support. The findings indicate that these teaching methods are jointly shaped by multiple factors and collectively promote the development of socio-emotional skills, linguistic and cognitive abilities, and inclusive attitudes.

**Conclusion:**

This study proposes a theory-driven framework of culturally inclusive teaching rooted in the Chinese context, offering actionable practical pathways for teacher professional development, curriculum design, and policy improvement. Meanwhile, analysis based on parent interviews clarifies the critical role of home–school collaboration mechanisms in supporting culturally inclusive practices. Together, the construction of a coordinated home–school educational ecosystem represents an important pathway for achieving the Sustainable Development Goal 4 (SDG 4) in early childhood education.

## 1 Introduction

With the acceleration of globalization and population mobility, early childhood education institutions have increasingly become microcosms of cultural diversity (Papakosma, 2023). Ages 3–6 constitute a critical period for young children's identity formation, social development, and the emergence of values. Therefore, promoting educational equity and cultural inclusivity at the kindergarten stage not only aligns with the core objectives of Sustainable Development Goal 4 (SDG 4) (UNESCO, 2016), but also serves as an essential foundation for cultivating global awareness, intercultural understanding, and inclusive attitudes (UNESCO, 2015). However, many studies suggest that culturally responsive education in practice continues to face challenges such as insufficient strategies, superficial implementation, and inadequate teacher preparation (UNESCO Global Education Monitoring Report Team, 2021; Veliz et al., 2025; Chen and Lai, 2024; Kehl et al., 2024).

From the perspective of Education for Sustainable Development (ESD), teachers play a key role in transforming macro-level inclusive learning into experiential and perceivable learning for young children (Cheng and Yu, 2022; Fischer et al., 2022; UNESCO, 2020). Chinese kindergartens are located at the intersection of national institutional arrangements, cultural traditions and global trends, providing a unique background for cultural diversity education. Therefore, research grounded in localized contexts is of critical importance for enhancing the quality of early childhood education in China. Against this background, the present study adopts a qualitative research design and enters authentic kindergarten teaching contexts to explore teachers' instructional practices in culturally diverse environments. The purpose of this study is to identify the core elements and dimensional structure of culturally inclusive teaching in Chinese early childhood education. Although culturally responsive education has received increasing attention, existing research has primarily focused on teachers' attitudes or isolated practices, with limited examination of how culturally inclusive teaching is systematically constructed and implemented in everyday kindergarten settings. By employing an integrative and context-sensitive analytical model grounded in the lived experiences of teachers and parents, this study addresses this gap in the literature. To this end, drawing on interviews with teachers and parents, the study employs a “context–method–outcome” analytical framework to address the following research questions. In this study, culturally inclusive teaching refers to the observable teaching practices and interaction strategies implemented by teachers in everyday kindergarten classroom settings.

RQ1: What core teaching methods do kindergarten teachers adopt in practice to promote culturally inclusive education, and how are these methods reflected in their reported teaching practices and classroom interactions?
RQ2: At the levels of young children, teachers, families, and kindergartens, which factors influence the selection and implementation of these teaching methods?
RQ3: What impacts do culturally inclusive teaching practices have on young children's development and teachers' professional growth?

## 2 Literature review

### 2.1 Definition of core concepts

#### 2.1.1 Culturally inclusive education

In the early childhood education stage for children aged 3–6, culturally inclusive education is regarded as a systematic process through which educators, via integrated practices such as curriculum design, learning environments, interactions, and assessment, acknowledge and actively respond to each child's cultural background, linguistic experiences, family practices, and identity characteristics (Shih, 2022; Mahadew, 2025). Its aim extends beyond the superficial representation of cultural diversity to the sustained creation of a learning community characterized by psychological safety, identity recognition, and a sense of belonging through ongoing instructional interactions, thereby supporting the social and emotional development of all young children within an equitable educational environment.

#### 2.1.2 Kindergarten teachers

In this study, kindergarten teachers refer to professional practitioners who provide systematic care and educational services for preschool children aged 3–6. Kindergarten teachers are not only the direct implementers of culturally inclusive education but also designers of learning environments and central bridges for home–school communication (Martínez-Rico et al., 2024). Teachers' cultural sensitivity, instructional decision-making capacity, and levels of reflective practice directly influence the quality with which culturally inclusive principles are enacted in everyday teaching.

#### 2.1.3 Culturally inclusive teaching methods

Culturally inclusive teaching methods refer to the specific strategies and behavioral patterns adopted by teachers in multicultural classrooms to promote educational equity and support the development of every preschool child. These methods encompass multiple dimensions, including curriculum design, environmental arrangement, teacher–child interactions, peer support, and home–school collaboration, emphasizing the systematic and contextualized nature of instructional strategies (Damjanovic and Ledford, 2024).

#### 2.1.4 Families as educational partners

Within the field of early childhood education, the role of families has gradually shifted from being mere providers of children's cultural backgrounds to active participants in the educational process. Existing studies indicate that families play a critical role in preschool children's identity development, value formation, and the shaping of learning experiences, a role that is particularly pronounced in culturally diverse contexts (Smith et al., 2020; Norheim et al., 2024; Otero-Mayer et al., 2025). Families are not passive recipients but provide contextual insights that inform classroom instruction by providing cultural knowledge, linguistic resources, and practical experiences.

In culturally inclusive education, the role of families extends beyond cultural representation to active participation in meaning-making and learning support. Accordingly, establishing collaborative and trust-based home–school relationships enables teachers to gain a deeper understanding of children's cultural identities, thereby adjusting instructional strategies and enhancing children's sense of belonging and psychological safety (Schwartz and Ragnarsdóttir, 2025; Mahadew, 2025). Moreover, from a sustainable development perspective, collaboration between families and kindergartens constitutes a core element in building a cooperative educational ecosystem. By fostering shared responsibility and co-constructed learning experiences, such collaboration can effectively enhance the long-term effectiveness and social sustainability of culturally inclusive education, particularly in early childhood education, where children's developmental trajectories are highly sensitive to interpersonal environments (Sheridan et al., 2010; Torres et al., 2015). These constructs were examined through teachers' reported practices, classroom observations, and records of parent interviews.

### 2.2 Research gaps and theoretical perspectives

Although culturally responsive teaching has developed a relatively well-established theoretical foundation at the K−12 level (Damjanovic and Ledford, 2024), empirical research on its practice in the kindergarten stage remains limited. Existing studies have largely focused on teachers' attitudes, cultural awareness, or isolated instructional activities, with insufficient holistic analysis of how teachers develop systematic teaching approaches within their everyday practices (Young and Young, 2023; Ng et al., 2022). Moreover, localized practice models applicable to China's multiethnic and increasingly internationalized contexts are still relatively underdeveloped (Vong and Wei, 2025). Unlike prior research that has primarily focused on the K−12 education stage or on individual characteristics, the present study adopts a practice-oriented perspective and examines culturally inclusive teaching as a relational and context-dependent process within Chinese kindergarten settings.

In recent years, the integration of ESD with early childhood education has emerged as a topic of growing international interest (Bascopé et al., 2019). Early childhood education represents a critical stage for cultivating young children's values and attitudes toward sustainable development, and teachers' classroom practices serve as a bridge for translating abstract sustainability goals into concrete everyday experiences (Cheng and Yu, 2022). In addition, research on social sustainability emphasizes that education systems play a foundational role in building harmonious and resilient societies by promoting equity, inclusion, and participation in cultural diversity (Tafese and Kopp, 2025).

## 3 Methods

### 3.1 Research design and methodological approach

This study adopted a qualitative research design (Creswell and Poth, 2018), with data collected via semi-structured interviews and observations. Qualitative research emphasizes the process of meaning construction and the interconnections among action, meaning, and interpretation (Charmaz, 2014), which aligns with the exploratory purpose of the present study and enables an in-depth understanding of kindergarten teachers' decision-making and processes in authentic teaching practices. The analytical process was grounded in a constructivist grounded theory approach, emphasizing the iterative interplay between data collection and data analysis, through which categories were co-constructed and progressively integrated into an explanatory model.

### 3.2 Participants

To ensure that the sample adequately reflected the diversity of kindergarten educational practices in China, 12 frontline kindergarten teachers were recruited from four kindergartens across different regions of China. These teachers varied widely in teaching experience (ranging from 2 to 12 years) and were distributed across four grade levels (C1–C4), thereby capturing diversity in instructional practices and classroom environments. In addition to teachers, eight parents were invited to participate in interviews to provide perspectives from the family context, particularly regarding identity formation, home–school collaboration, and influences within multicultural kindergarten environments. The supplementary insights provided by parent participants enriched the depth of data triangulation. All participants provided written informed consent. To protect privacy, all participants were presented using anonymized identification codes (Table 1). No data were directly collected from minors. Classroom observations focused exclusively on teachers' instructional practices, and no identifiable information related to children was recorded. Apart from teaching experience and class level, no additional demographic information was collected, as the focus of this study was on teaching practices rather than participant characteristics.

**Table 1:** Participant profile.

**Code**	**Teaching experience**	**Class**
PT1	8 years	C1
PT2	6 years	C1
PT3	8 years	C1
PT4	12 years	C2
PT5	3 years	C2
PT6	9 years	C2
PT7	9 years	C3
PT8	8 years	C3
PT9	2 years	C3
PT10	7 years	C4
PT11	5 years	C4
PT12	4 years	C4
PP1	-	C1
PP2	-	C1
PP3	-	C2
PP4	-	C2
PP5	-	C3
PP6	-	C3
PP7	-	C4
PP8	-	C4

PT, teacher participants; PP, parent participants. For parent participants (PP), teaching experience is not applicable and therefore indicated as “–”.

### 3.3 Data collection

To ensure data triangulation and to construct a theoretical model based on multiple perspectives, data were collected from four complementary sources: (1) semi-structured interviews with teachers, focusing on instructional methods and decision-making contexts (Supplementary Table S1); (2) semi-structured interviews with parents, focusing on family contextual factors and observations of children's developmental outcomes (Supplementary Table S4); (3) non-participant classroom observations, focusing on the implementation of teaching methods; and (4) the collection of relevant documents, primarily teaching plans, as Supplementary data for verification purposes. Detailed interview protocols and observation foci are provided in Supplementary Tables S1, S4.

### 3.4 Data analysis

**Figure 1:**
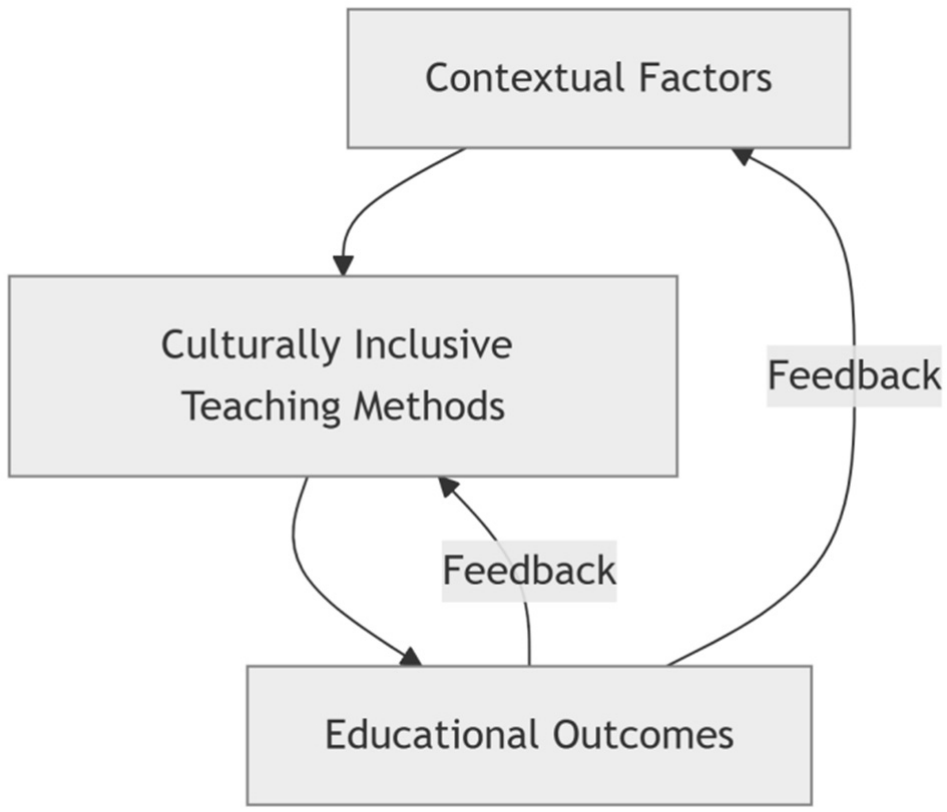
The “context–methods–outcomes” theoretical model of culturally inclusive teaching practices.

Data analysis was conducted through a systematic coding process comprising four stages: open coding, axial coding, selective coding, and theoretical integration (Table 2). The coding process was jointly conducted by two researchers, both of whom possessed knowledge of constructivist grounded theory. An initial set of interview transcripts was independently coded and discussed to refine the coding framework. Subsequently, this framework was applied to the entire dataset and implemented through repeated negotiation. Teacher interviews (PT), parent interviews (PP), non-participant classroom observation records (Po), and related documentary materials (Pd) were integrated into a single corpus. Through selective coding, three overarching core categories were extracted from the teacher data: culturally inclusive teaching methods, influencing factors, and educational outcomes (Supplementary Tables S2, S3). Analysis of the parent interview data yielded five core categories related to family involvement and cognitive dimensions (Supplementary Table S5). During the theoretical integration stage, categories derived from parent data were consolidated into three overarching core categories, thereby deepening the understanding of family-related factors and educational outcomes (Supplementary Table S6). The final theoretical model is presented in Figure 1. Data collection and analysis were conducted iteratively until theoretical saturation was reached, defined as the point at which no new themes or categories emerged during the later stages of coding.

**Table 2:** Example of axial coding: from open coding to major categories.

**Coding stage**	**Core task**	**Key analytical actions**	**Main output**
Open coding	Initial fragmentation and conceptualization of raw data	Line-by-line coding; labeling meaningful units; constant comparison to cluster similar concepts	158 initial concepts consolidated into 24 preliminary categories
Axial coding	Establishing relationships among categories	Integrating categories through the “conditions–actions/interactions–consequences” logic; grouping conceptually related categories	24 preliminary categories merged into 12 higher-level categories
Selective coding	Identifying the core category and building the storyline	Comparing and abstracting major categories to extract an overarching core category and clarify its logical linkages	Three core categories identified: Culturally Inclusive Teaching Methods, Influencing Factors, and Educational Outcomes
Theoretical integration	Constructing the explanatory model	Synthesizing core categories through theoretical coding to form a coherent analytic framework	Development of a “Context–Methods–Outcomes” dynamic model explaining practice mechanisms

To enhance trustworthiness, this study followed the qualitative validity criteria proposed by Lincoln and Guba (1985) and Enworo (2023). Strategies including prolonged field engagement, in-depth interviews, data triangulation, member checking, peer debriefing, and researcher reflexivity were employed to reduce researcher bias and strengthen analytic credibility.

## 4 Findings

### 4.1 Framework of core teaching methods

**Figure 2:**
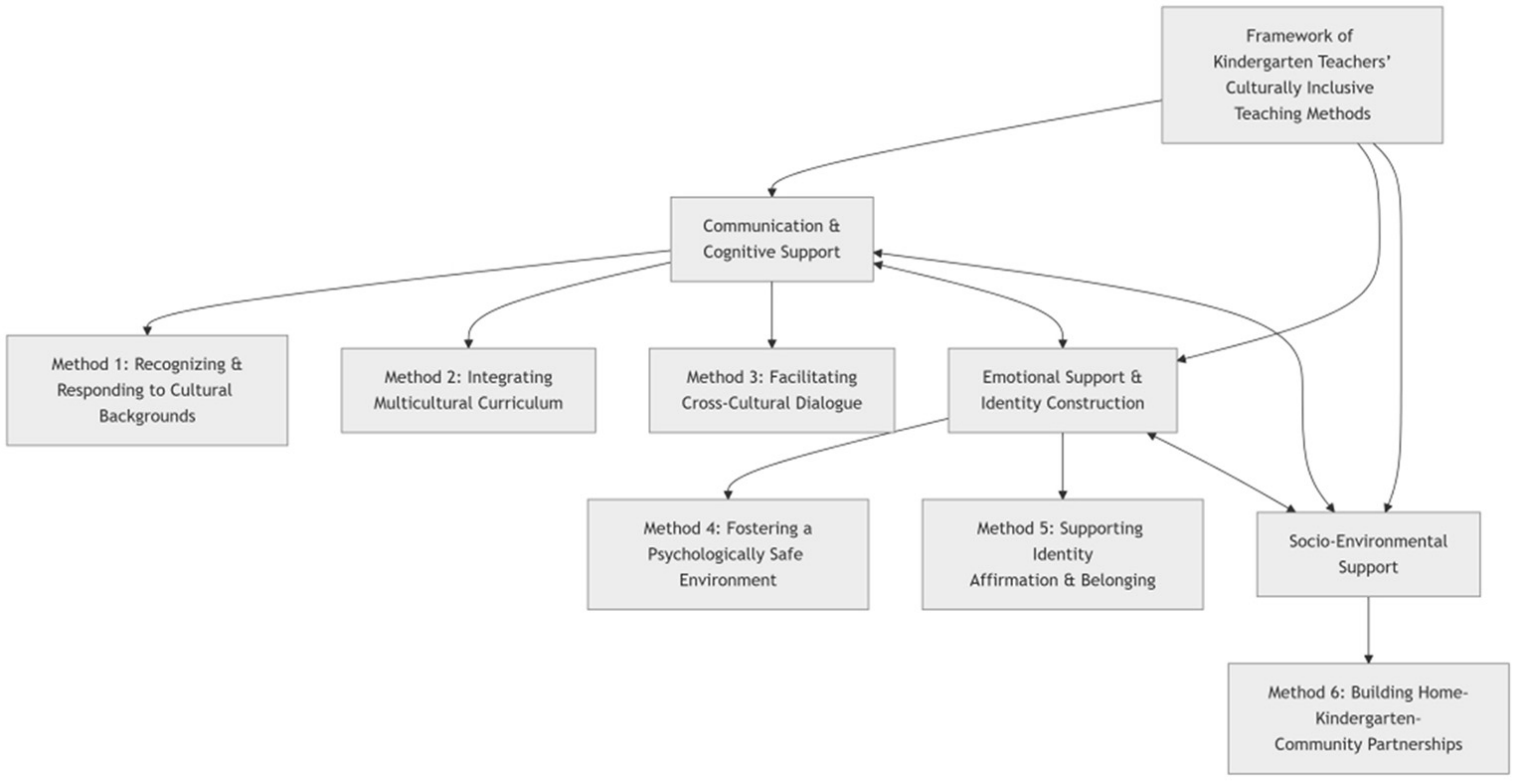
Framework of kindergarten teachers' culturally inclusive teaching methods.

The analysis identified six recurrent teaching methods reported by kindergarten teachers in multicultural instructional practices. Although analytically distinguished, these six teaching methods form an interrelated system through three overarching dimensions, jointly shaping culturally inclusive teaching practices. These methods do not operate independently; rather, they are interrelated within an overarching framework comprising three dimensions—communicative and cognitive support, emotional support and identity construction, and social–environmental support (Figure 2).

#### 4.1.1 Communicative and cognitive support dimension

##### 4.1.1.1 Method 1: recognizing and responding to cultural backgrounds

Teachers provide frameworks for cognitive and language development by engaging children in in-depth reading and discussion of multicultural picture books, deliberately introducing new vocabulary and sentence structures in project-based activities, and encouraging children—especially those from different linguistic backgrounds—to express themselves around cultural themes. As Teacher T5 noted, “Around the theme of ‘home,' we read many picture books about living environments in different countries. The children were very curious, and I guided them to compare, describe, and express their ideas to one another” (Po-C2).

##### 4.1.1.2 Method 2: integrating a multicultural curriculum

Teachers design role-play areas with cross-cultural themes, guide children to conduct cultural exploration projects, and enable them to develop cultural understanding through direct experience and creative expression via drawing, handicrafts, music, and dance. Teacher T11 described a project themed around the “Silk Road,” in which children worked in groups to study different products and cultures and ultimately organized a small exhibition to present their learning to children from other classes (Pd).

##### 4.1.1.3 Method 3: facilitating cross-cultural dialogue

Teachers employ body language, facial expressions, modeling, and visual cues as instructional supports, while continuously observing children's behaviors and emotions to interpret the strategies and feelings underlying nonverbal expressions. Teacher T7 explained, “For children who are new to the kindergarten, I take the initiative to communicate through action-based demonstrations and expressive facial cues. Once the relationship becomes closer and more familiar, the children will actively pull my hand and use gestures to tell me what they need” (Po-C3).

#### 4.1.2 Emotional support and identity construction dimension

##### 4.1.2.1 Method 4: fostering a psychologically safe environment

Teachers attend to emotional issues that may arise from cultural adaptation or identity-related confusion. Through one-on-one communication and shared picture book reading, they help children recognize and express their emotions and guide them to cope with conflicts and challenges in positive ways. As Teacher T10 described, “When a child feels anxious about pronunciation, I first soothe the child's emotions and affirm their uniqueness, and then use stories to guide the whole class to understand that everyone is different and deserves understanding” (Po-C4).

##### 4.1.2.2 Method 5: supporting identity affirmation and a sense of belonging

Teachers treat children's family cultures as curricular resources and integrate them into classroom theme walls, activities, and collective instruction. In addition, different cultural customs and festivals are introduced with an egalitarian and objective stance, avoiding the framing of cultures as “other” or “deviant.” Teacher T1 noted, “I combined the themes of ‘transportation' and ‘my family' for sharing activities, which the children found very interesting. I also noticed that children's learning styles, interests, and abilities are influenced by their cultural backgrounds and personal experiences. Therefore, I provide hands-on materials, allow children to choose different ways to demonstrate their understanding, and use small-group formats to ensure that every child can experience success.” Teacher T6 further explained that in the activity themed “Beautiful Patterns,” multiple materials such as ink painting, paper cutting, and collage were provided, enabling children to present work they were satisfied with using methods they were skilled in and enjoyed (Pd).

#### 4.1.3 Social–environmental support dimension

##### 4.1.3.1 Method 6: building home–kindergarten–community partnerships

Teachers intentionally design tasks that require collaborative effort, thereby fostering communication and a sense of collectivity among children. When conflicts arise due to differing opinions or behavioral habits, teachers treat these moments as valuable opportunities for social learning, guiding children to listen to one another's perspectives, understand each other's feelings, and jointly negotiate solutions. Teacher T12 described, “When two children had different opinions about the rules of a game, I called them aside and asked each of them to express their thoughts and feelings. In the end, they negotiated a new rule themselves.”

In addition, teachers proactively establish egalitarian and trust-based relationships with families. Through regular home visits, parent open days, workshops, and class parent groups, teachers maintain ongoing communication with parents and actively invite them to participate in curriculum design, while also integrating community resources into the kindergarten. Teacher T9 noted that a Tibetan mother of a child in the class was invited to teach a simple “Tibetan dance,” which the children greatly enjoyed. Parents confirmed this practice and provided positive feedback. As Parent PP5 stated, “When I was invited to teach Tibetan dance, I felt that the kindergarten truly valued the integration and recognition of different ethnic cultures.” Such bidirectional interactions further strengthened collaborative home–kindergarten relationships.

### 4.2 Influencing factors of culturally inclusive teaching methods

**Figure 3:**
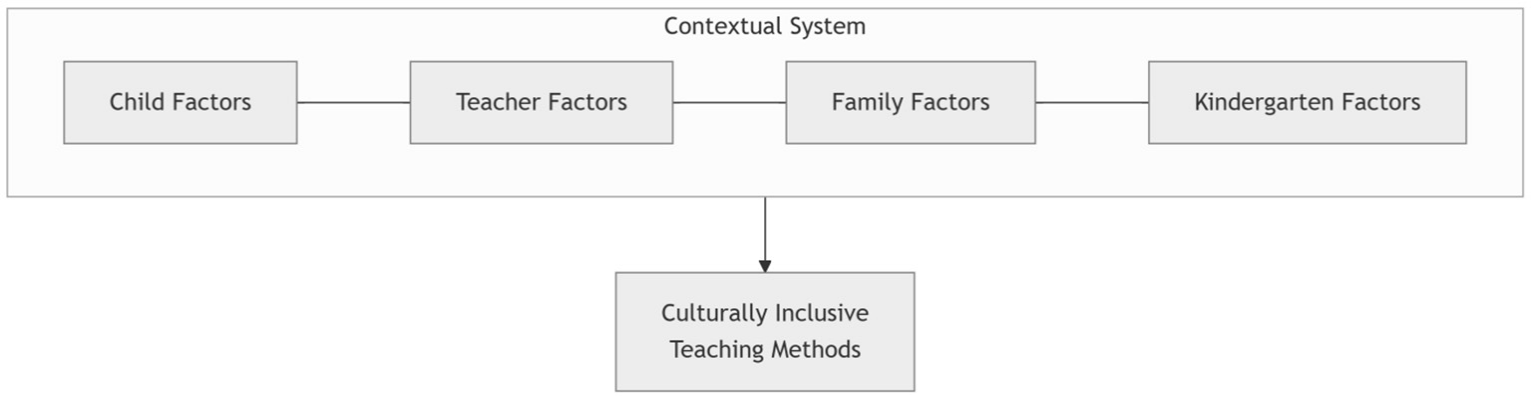
Framework of factors influencing culturally inclusive teaching methods.

This study found that culturally inclusive teaching methods do not operate in isolation but are jointly shaped by multi-level systemic factors, including young children, teachers, families, and kindergartens (see Figure 3). These factors are interwoven and influence teachers' instructional decisions and practice pathways through mechanisms such as contextual constraints, value orientations, and resource support.

#### 4.2.1 Child factors

##### 4.2.1.1 Cognitive and language development levels

Young children's age-related characteristics, language expression abilities, and levels of comprehension directly influence teachers' choices of instructional content and communication approaches. Teacher T2 noted that, based on teaching experience and classroom interaction observations, teachers generally perceive older preschool children as having stronger language comprehension and discussion abilities. Consequently, teachers are more inclined to introduce relatively abstract themes, such as “fairness and bias,” with older children, whereas younger children require greater reliance on concrete behaviors and guided language support (Po-C1/C2).

##### 4.2.1.2 Social development and peer relationships

Patterns of interaction among children, their conflict resolution abilities, and their degree of acceptance within peer groups serve as important references for teachers when determining how to guide peer interactions. Teacher T4 mentioned that in everyday classroom interactions, some children display withdrawn behaviors when engaging with unfamiliar peers. In such cases, teachers typically first create experiences of acceptance through shared play activities and then gradually guide children to express their personal ideas (Po-C2).

##### 4.2.1.3 Temperament and cultural expression

Children's temperament (e.g., extroversion or introversion), levels of expressive confidence or hesitation, and behavior patterns shaped by their cultural backgrounds require teachers to adopt differentiated response strategies. Teacher T8 described that in her teaching practice, teachers sometimes encounter children who are relatively introverted yet show strong interest in specific cultural themes. In response, teachers tend to initially arrange small-group sharing activities to help children gradually build confidence in expression before transitioning to whole-class communication.

#### 4.2.2 Teacher factors

##### 4.2.2.1 Educational beliefs and cultural perspectives

Whether teachers deeply uphold the values of equity and inclusion and possess the capacity for cultural self-reflection constitutes a core driving force behind their instructional choices. Teacher T1 stated, “I have always believed that education is about allowing every child to shine and find their own value. When I see a child being marginalized, I cannot stand by and do nothing.”

##### 4.2.2.2 Professional knowledge and skills

Teachers' mastery of early childhood developmental principles, multicultural theories, and specific instructional strategies directly determines the depth and breadth of their practices. Teacher T12 explained, “If I do not understand children's developmental characteristics, it is difficult to judge whether they can comprehend certain cultural concepts. I also need to keep learning new teaching approaches, such as using project-based activities to guide children in exploring culture.”

##### 4.2.2.3 Personal experiences and reflective capacity

Teachers' own developmental experiences and cross-cultural encounters, as well as the frequency and depth of their reflective practices, directly influence their cultural sensitivity and pedagogical innovation. Teacher T7 noted, “I experienced confusion caused by cultural differences when I was young, so I pay particular attention to children's feelings. After each activity, I record the cultural situations I encounter and reflect on whether I can do better next time” (Pd).

#### 4.2.3 Family factors

##### 4.2.3.1 Parenting beliefs and cultural identity

Parents' attitudes toward their own cultures and toward cultural diversity, as well as their expectations of kindergarten education, directly influence their willingness and modes of participation in home–kindergarten collaboration. Teacher T10 noted that some parents strongly hope their children will retain their ethnic traditions and therefore actively cooperate with teachers by providing materials for classroom use, whereas other parents prefer their children to “integrate into the mainstream” as quickly as possible. Parent interviews confirmed these divergent beliefs. Parent PP5 explicitly expressed a strong desire for cultural transmission, stating a willingness to teach children Tibetan dance: “This is not just about showing it, but about telling children that our culture is beautiful and something to be proud of.” In contrast, Parent PP6 emphasized cultural adaptation, expressing a stronger preference for children to quickly master Mandarin and a reluctance to highlight practices from other regions. Such diversity requires teachers to adopt differentiated strategies for home–kindergarten communication.

##### 4.2.3.2 Quality of home–school communication

Whether open and trust-based communication channels are established between parents and teachers is crucial for effective collaboration in supporting children's development. Teacher T11 noted, “Some parents are not very willing to express themselves at first. I take the initiative to contact them and gradually build mutual trust. Once we become familiar with each other, they share many details about family culture, which is particularly helpful for teaching.” Parents' experiences further confirmed the importance of communication quality. Parent PP2 stated, “The teacher often shares children's drawings of different ethnic patterns and asks me whether they are accurate. I feel respected and am more willing to become involved in kindergarten teaching.” Such positive communication effectively enhances both the willingness and depth of family participation (Pd).

#### 4.2.4 Kindergarten factors

##### 4.2.4.1 Kindergarten culture and leadership support

Whether the overall atmosphere of a kindergarten is open and inclusive or conservative and closed, and whether the principal regards cultural inclusivity as a core educational philosophy and provides corresponding resource support, can either motivate or constrain teachers' practices. Teacher T5 noted, “The principal is very supportive and has specifically established a ‘multicultural resource bank,' which we can access at any time. This has provided great convenience for our work.”

##### 4.2.4.2 Curriculum system and professional development activities

Whether the kindergarten curriculum is rigid and uniform or encourages generative development, and whether related teaching and research activities are organized to promote teachers' professional growth, provide an institutional platform for instructional practice. Teacher T12 described that a cultural education–themed exchange meeting is held every month, during which teachers share cases and experts provide guidance, giving them clearer direction for culturally responsive teaching (Pd).

##### 4.2.4.3 Resource allocation and policy environment

Teacher–child ratios, the availability of material resources within kindergartens, and policy orientations of local educational authorities constitute the broader structural conditions shaping teachers' practices. Teacher T10 noted, “If class sizes are too large or materials are insufficient, it becomes difficult to carry out cultural exploration activities in depth. When local policies provide resources or training opportunities, the outcomes are often much better” (Pd).

### 4.3 Outcomes of implementing culturally inclusive teaching

**Figure 4:**
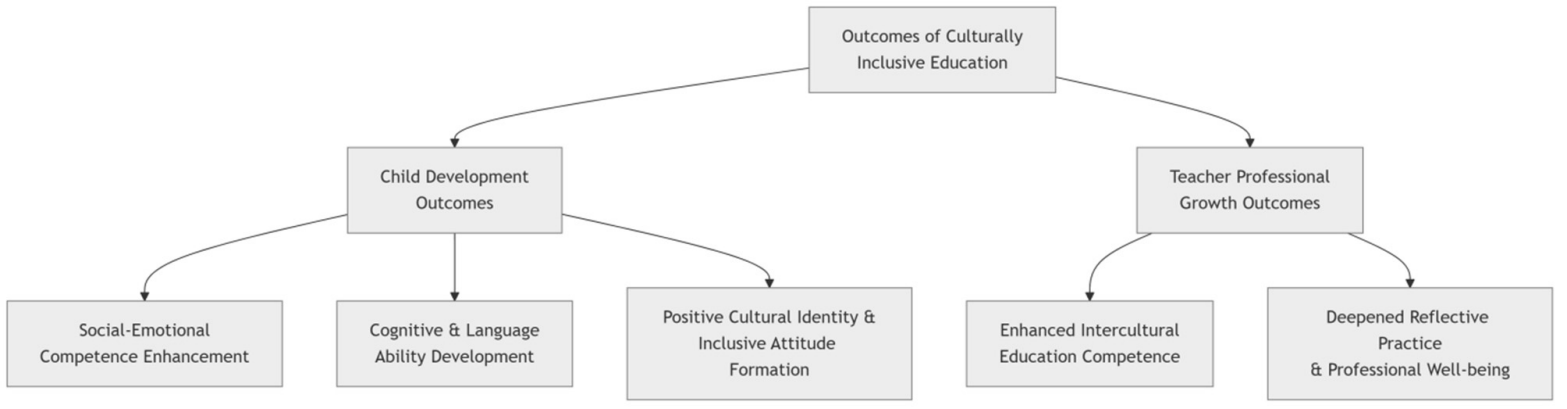
Outcomes of culturally inclusive education.

The sustained implementation of culturally inclusive education was associated with reported positive outcomes at two levels: young children's development and teachers' professional growth (see Figure 4). These outcomes are reflected not only in the enhancement of individual competencies but also in deeper improvements in interaction quality and value orientations within the educational ecosystem.

#### 4.3.1 Developmental outcomes for young children

##### 4.3.1.1 Enhancement of socio-emotional competence

Children demonstrated stronger self-esteem and confidence, improved abilities to recognize and regulate their emotions, and markedly enhanced empathy and prosocial behaviors. Teacher T10 noted that a child who had previously been mocked for pronunciation later became the class's “little language recitation star,” proactively introducing their hometown to classmates and becoming notably confident and outgoing. Parents corroborated this observation; Parent PP1 reported that the child had once felt timid because of an accent but now confidently shares songs from their hometown with peers, indicating a significant increase in resilience.

##### 4.3.1.2 Development of cognitive and language abilities

Within rich and meaningful learning contexts, children's language expression became more accurate, and their openness of thinking and creativity were stimulated. Teacher T1 described that during project-based inquiry, children actively sought information by consulting books and asking teachers and parents questions in order to clarify issues, a level of learning initiative and depth that she found surprising. Parents confirmed this growth in autonomous learning. Parent PP3 noted that after completing the “Silk Road” project at kindergarten, the child now frequently asks about different countries and even creates trade maps at home to present their understanding.

##### 4.3.1.3 Formation of positive cultural identity and inclusive attitudes

As children developed positive and healthy cultural self-identities, they also exhibited curiosity, respect, and appreciation toward different cultures, and began to acquire the ability to view issues from multiple perspectives. Teacher T6 explained that when children now encounter differing opinions or habits among classmates, their first response is no longer to argue but to ask curiously, “Why is that?” and they often identify underlying reasons. Parents confirmed this shift. Parent PP7 stated that the child now proudly explains the family's ethnic minority New Year calendar to friends, viewing differences not as barriers but as interesting facts worth sharing.

#### 4.3.2 Outcomes for teachers' professional growth

##### 4.3.2.1 Significant enhancement of intercultural educational competence

Teachers demonstrated marked improvements in cultural sensitivity, curriculum development and integration capacities, abilities to observe and interpret children's behaviors, and skills in communicating and collaborating with families. Teacher T11 noted, “Engaging in these practices is itself a continuous learning process for me. I now approach issues from broader perspectives and consider matters more comprehensively when designing activities.”

##### 4.3.2.2 Deepening of reflective practice awareness and professional well-being

Teachers became more accustomed to examining their own educational practices and potential biases, shifting their roles from passive implementers of curricula to active reflective practitioners and researchers. Successful educational practices fostered a strong sense of professional achievement and meaning. Teacher T8 described, “When I see that, because of my efforts, a child—or even the atmosphere of an entire class—has changed in a positive way, that sense of accomplishment is irreplaceable and makes me feel that this work is especially meaningful.”

## 5 Discussion

### 5.1 Synergistic mechanisms of teaching methods

**Figure 5:**
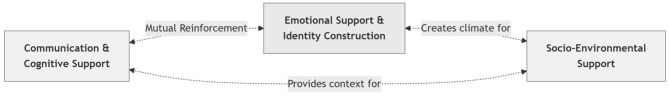
Synergistic interaction mechanism of culturally inclusive teaching methods.

These six categories of teaching methods and their three overarching dimensions can be interpreted as constituting an interdependent and synergistic system that explains how culturally inclusive teaching is implemented within kindergarten settings (Figure 5). This system aligns closely with established mechanisms of psychosocial development and cultural learning in early childhood education (Shih, 2022).

#### 5.1.1 Communicative and cognitive support

This dimension extends prior discussions of communicative support and cognitive support by emphasizing how language scaffolding and embodied learning are adapted to culturally diverse kindergarten environments (Papakosma, 2023; Ng et al., 2022). Without this foundational support, emotional acceptance and identity formation would lack rational grounding (Gay, 2018; Hammond, 2014). In practice, this dimension is reflected in Methods 1–3. For example, PT5 facilitated discussions through multicultural picture books, and PT11 designed the “Silk Road” project. Non-participant classroom observations (Po-C2) further confirmed the effectiveness of these methods in stimulating children's abilities to compare, describe, and articulate their ideas.

#### 5.1.2 Emotional support and identity construction

From a comparative perspective, this dimension resonates with international research on emotional safety and identity formation, while also highlighting processes of cultural positioning within Chinese kindergarten contexts (Chen and Lai, 2024). Such a sense of safety and positive self-awareness serves as an internal driving force enabling young children to engage confidently in cross-cultural exploration and interaction, transforming cognitive-level “understanding” into affective-level “identification.” This process aligns with global trends in early childhood education that emphasize fostering positive cultural identity among young children (UNESCO Global Education Monitoring Report Team, 2021). Teachers' Methods 4–5 directly aim to establish this safe environment. Parents provided critical external validation of these processes: PP1 observed that the child became notably confident and outgoing after experiencing acceptance, while PP7 described the child's shift from “concealing” to “proudly” explaining their family culture, marking the genuine transformation of cognitive “understanding” into emotional “identification.”

#### 5.1.3 Social–environmental support

Positive collaborative interactions and in-depth home–kindergarten–community cooperation provide sustained external support and authentic practice contexts for young children's cultural adaptation and inclusive behaviors. Children practice inclusivity within collective settings and consolidate their learning outcomes through continuous experiences across family and community contexts (Martínez-Rico et al., 2024; Damjanovic and Ledford, 2024). These three dimensions are interconnected and mutually reinforcing, reflecting the interactive influences of multi-level systems on children's development emphasized in ecological systems theory (Cheng and Yu, 2022), thereby forming a complete theoretical model of a spiraling, upward synergistic effect. Method 6 serves as the core of this dimension. PT9 proactively invited parental participation, PP5 reported the resulting sense of value and trust derived from involvement, and classroom observations (Po-C3) documented the enjoyment and engagement evident during such collaborative activities.

Accordingly, the three dimensions do not operate independently but are interrelated and mutually strengthening, a pattern confirmed through integrative analysis of teacher interviews (PT), parent interviews (PP), and classroom observations (Po). This phenomenon reflects the emphasis of ecological systems theory on interactions among multi-level systems (Cheng and Yu, 2022). By integrating PT, PP, Po, and Pd, the present study provides a detailed account of how this spiraling synergistic effect is concretely formed within culturally inclusive kindergarten practices in the Chinese context. The “context–method–outcome” model proposed in this study not only aligns with international frameworks of culturally responsive teaching, but also extends prior research by explicitly articulating contextual conditions and home–school collaboration within Chinese kindergarten settings. The following discussion focuses on interpreting these findings in relation to existing literature and theoretical perspectives, rather than reiterating the empirical results.

### 5.2 Practical implications and pathways toward sustainable development

#### 5.2.1 Teacher level

##### 5.2.1.1 Cultural awareness and reflective practice

The findings indicate that the extent to which teachers engage in reflective examination of their cultural perspectives is closely associated with the implementation of culturally inclusive teaching practices. This process is critical for the development of cultural awareness and reflective practice (Young and Young, 2023; Kehl et al., 2024).

##### 5.2.1.2 Culturally inclusive teaching practice

The findings indicate that flexible curriculum generation and project-based learning are key features of the culturally inclusive teaching practices identified in this study. This approach is consistent with internationally emphasized systematic professional development pathways for culturally responsive teaching (Damjanovic and Ledford, 2024; Mahadew, 2025).

##### 5.1.1.3 Professional learning communities

The analysis emphasizes that professional learning communities constitute an important contextual condition supporting teachers' sustained engagement in culturally inclusive teaching practices. Professional communities have been shown to be important mechanisms for promoting teachers' multicultural knowledge and agency (Martínez-Rico et al., 2024).

#### 5.2.2 Kindergarten level

##### 5.2.2.1 Leadership vision and inclusive culture

The findings indicate that leadership vision and an inclusive institutional culture at the kindergarten level play a central role in shaping teachers' culturally inclusive teaching practices, which is consistent with prior research emphasizing the influence of school cultural ecology (Papakosma, 2023).

##### 5.2.2.2 Institutionalized support and professional development

The findings indicate that institutionalized support mechanisms (such as school-based teacher professional development and resource provision) are closely associated with the sustainability of culturally inclusive teaching practices. Systematic institutional support has been shown to significantly enhance teachers' culturally responsive teaching practices (Veliz et al., 2025).

##### 5.2.2.3 Home–school–community co-construction

The findings indicate that home–school–community collaboration is most effective when it moves beyond passive participation toward more dialogic and co-constructed forms of engagement. This shift enables home–school–community collaboration to move from “passive participation” toward “egalitarian partnership.” Deep engagement of families and communities is widely regarded as a critical external resource for culturally inclusive education (Martínez-Rico et al., 2024).

### 5.3 Limitations and future directions

Although this study reveals the relational structure among “context–method–outcome” within the ecosystem of culturally inclusive education, several limitations should be acknowledged. First, this study mainly involved kindergartens located in East China and North China; therefore, caution is required when generalizing the findings across regions. Second, although this study systematically incorporated dual perspectives from teachers and parents, it did not include the direct voices and lived experiences of young children themselves. Third, the culturally inclusive teaching model was primarily constructed from the teacher perspective and has not yet systematically integrated multiple perspectives from young children, parents, and kindergarten administrators. Fourth, as a cross-sectional qualitative study, this research elucidated the relational structure among “context–method–outcome,” but it cannot establish causal relationships or identify long-term effects.

To address the limitations noted above, future research should build upon the present study in the following ways. First, future studies should expand the sampling scope to include kindergartens from a wider range of regions in order to enhance the generalizability of the findings. Second, research designs should adopt age-appropriate methods that foreground children's perspectives. Third, multi-stakeholder research designs should be employed to more comprehensively capture the interaction processes involved in culturally inclusive practices. Fourth, future research may adopt longitudinal and mixed-methods designs to more accurately trace the development and evolution of these mechanisms in culturally inclusive teaching practices over time.

## 6 Conclusion

This study constructed a “context–method–outcome” theoretical model of culturally inclusive educational practices among kindergarten teachers in China. The model was developed through multi-source data triangulation based on teacher interviews, parent interviews, and classroom observations.

### 6.1 Contextual influences level

The study clarifies how four categories of factors—young children, teachers, families, and kindergartens—interactively shape teachers' selection, implementation, and innovation of culturally inclusive teaching methods through pathways such as value orientations, professional competencies, home–school relationships, kindergarten culture, and resource and policy conditions, thereby revealing a multi-level connective structure underlying micro-level classroom practices. In addition, analysis of parent interview data demonstrates that families are not merely background factors but active collaborators whose cultural identities and communication quality directly influence instructional decision-making.

### 6.2 Educational outcomes level

The findings indicate that culturally inclusive education promotes young children's socio-emotional development, cognitive and language development, positive cultural identity, and inclusive attitudes, while also enhancing teachers' intercultural educational competence, reflective awareness, and sense of professional accomplishment. Furthermore, it fosters more stable and egalitarian home–school collaborative relationships, which constitute an important educational outcome in their own right. These findings provide practical guidance for kindergarten teachers, school administrators, and policymakers, with the aim of advancing early childhood education that integrates cultural inclusivity with educational equity.

Therefore, the empirically grounded “context–method–outcome” model proposed in this study, together with the articulated pathways of home–school collaboration, provides valuable theoretical guidance and a practical framework for advancing the SDG 4 of equitable, inclusive, and quality education in early childhood education across diverse cultural contexts.

## Data Availability

The original contributions presented in the study are included in the article/Supplementary material, further inquiries can be directed to the corresponding author.
