# Supplementary material for: Culturally inclusive teaching in diverse classroom settings in Chinese kindergartens: a qualitative “context–methods–outcomes” model
Source: Front Psychol. 2026 Feb 12;17:1772144. doi: 10.3389/fpsyg.2026.1772144 (PMC12935598; doi:10.3389/fpsyg.2026.1772144)
Supplement: Supplementary file 1 [file Table_1.docx]

**Supplementary Table S1. Semi-Structured Interview Protocol**

| **No.** | **Core Interview Question (Refined English Version)** |
| --- | --- |
| 1 | How would you describe the cultural backgrounds of children in your class? Please give examples of how you design activities to support cultural interaction and inclusion. |
| 2 | How do you respond to and communicate with children from different cultural or linguistic backgrounds? Please describe specific strategies and examples. |
| 3 | In what ways do you integrate multicultural elements into your curriculum and daily learning environment? Please share relevant practices. |
| 4 | How do you handle behavioral or emotional issues related to cultural differences? How do you collaborate with families from diverse backgrounds? |
| 5 | What major challenges have you encountered in implementing culturally inclusive practices? What kinds of support or professional development do you think are needed? |
| 6 | What value do you believe cultural diversity brings to children’s development and your own professional growth? Do you have any additional reflections or suggestions? |

**Supplementary Table S2. Core Teaching Methods Identified through Selective Coding**

| **Core Method Category** | **Refined Description** |
| --- | --- |
| 1. Linguistic and Cognitive Scaffolding | Providing comprehension and expression support through multilingual materials, guided conversations, and contextual vocabulary learning. |
| 2. Nonverbal and Environmental Communication | Using gestures, facial expressions, visual cues, and culturally inclusive environmental design to facilitate communication and convey inclusiveness. |
| 3. Play-Based and Project-Based Multicultural Learning | Designing cross-cultural role-play, thematic inquiry projects, and creative arts activities that allow children to experience, express, and interpret cultural meanings. |
| 4. Curriculum Integration of Cultural Elements | Embedding children’s family cultures, festivals, traditions, and daily practices into curriculum activities to ensure representation and cultural relevance. |
| 5. Emotional Support and Climate Building | Addressing emotional issues through empathy, guidance, and story-based discussions to create a safe, accepting, and trustful classroom atmosphere. |
| 6. Differentiated and Collaborative Learning Strategies | Using flexible grouping, diverse materials, and peer collaboration strategies to support children’s varied learning needs and promote intercultural peer interaction. |

**Supplementary Table S3. Final Core Categories and Constituent Elements**

| **Core Category** | **Refined Description** |
| --- | --- |
| Culturally Inclusive Teaching Method System | A coherent set of six interrelated strategies guiding teachers’ cultural inclusion practices in daily classroom activities. |
| Influencing Factors of Culturally Inclusive Teaching | A multi-level set of contextual conditions including children's individual characteristics, teachers’ beliefs and competencies, family attitudes and engagement, and institutional policies and environmental support. |
| Educational Outcomes of Culturally Inclusive Practice | Outcomes include enhanced social, cognitive, and emotional development for children, and improved intercultural awareness, reflective capacity, and professional growth for teachers. |

**Supplementary Table S4. Semi-Structured Interview Protocol for Parents**

| **No.** | **Core Interview Question (English Version)** |
| --- | --- |
| 1 | How would you describe your family’s cultural background and the cultural environment at home? How do you share this with your child and the kindergarten? |
| 2 | What cultural or festive activities organized by the kindergarten have you participated in or are aware of? How do you evaluate these activities? |
| 3 | How does the kindergarten communicate with you regarding cultural inclusion or multicultural activities? Please describe your experience with teacher–parent communication. |
| 4 | Have you ever provided cultural resources (e.g., stories, artifacts, skills) to the kindergarten? If so, please describe the process and your feelings about it. |
| 5 | What changes have you observed in your child’s attitudes or behaviors toward different cultures since attending kindergarten? Please provide examples. |
| 6 | What suggestions do you have for the kindergarten to better support cultural inclusion or to collaborate with families like yours? |

**Supplementary Table S5. Core Family Engagement and Perception Categories Identified through Selective Coding**

| **Core Category** | **Refined Description** |
| --- | --- |
| 1. Family Cultural Identity Expression | Willingness and modes through which families share their cultural heritage, traditions, and values with the kindergarten and their children. |
| 2. Participation in Kindergarten Cultural Activities | Levels and forms of involvement in multicultural events, curriculum co-construction, and resource provision initiated by the kindergarten. |
| 3. Quality of Home–Kindergarten Communication | Perceptions of communication frequency, clarity, trust, and mutual respect between parents and teachers regarding cultural matters. |
| 4. Observed Child Development in Cultural Contexts | Parents’ narratives regarding changes in their children’s cultural awareness, empathy, language use, and social interactions in diverse settings. |
| 5. Expectations and Suggestions for Inclusive Collaboration | Parents’ expressed needs, hopes, and practical recommendations for enhancing culturally inclusive practices and home–kindergarten partnerships. |

**Supplementary Table S6. Final Core Categories Integrating Parent Perspectives**

| **Core Category** | **Refined Description** | **Illustrative Quote from Parent Data** |
| --- | --- | --- |
| Family as Cultural Resource & Collaborator | Families actively contribute cultural knowledge, materials, and lived experiences to the kindergarten curriculum, transitioning from passive recipients to active co-constructors. | I taught the children a Mongolian dance and brought traditional costumes. The teacher filmed it and used it in class for weeks. I felt my culture was valued. |
| Home–Kindergarten Trust and Communication Dynamics | The establishment of open, respectful, and consistent communication channels that facilitate mutual understanding and collaborative problem-solving around cultural inclusion. | The teacher often sends photos and explains how my child’s cultural background is included in activities. It makes me feel seen and trusted. |
| Parent-Perceived Child Outcomes in Multicultural Settings | Parents’ observations of growth in their children’s socio-emotional skills, identity affirmation, intercultural curiosity, and adaptive behaviors within diverse peer groups. | My son now proudly explains our Tibetan New Year to his friends. He used to hide that part of himself. |
| Contextual Enablers & Barriers to Family Engagement | Factors at the family, teacher, and kindergarten levels that either facilitate or hinder meaningful parental involvement in cultural inclusivity efforts. | I’d love to participate more, but the activities are often during work hours. Maybe weekend cultural fairs would help. |
